# Brainstem auditory evoked potentials in patients with primary Sjögren’s syndrome without central nervous system involvement

**DOI:** 10.1007/s10067-020-05344-5

**Published:** 2020-08-18

**Authors:** Marta Waliszewska-Prosół, Agata Sebastian, Piotr Wiland, Sławomir Budrewicz, Edyta Dziadkowiak, Maria Ejma

**Affiliations:** 1 Department of Neurology, Wrocław Medical University, Borowska 213, 50-556 Wrocław, Poland; 2 Department of Rheumatology and Internal Diseases, Wrocław Medical University, Borowska 213, 50-556 Wrocław, Polska

**Keywords:** Brainstem auditory evoked potentials, Brainstem function, Evoked potentials, Primary Sjögren’s syndrome

## Abstract

**Objectives:**

Primary Sjögren’s syndrome (pSS) is an autoimmune, multisystem exocrinopathy characterized by dysfunction of the exocrine glands. Central nervous system (CNS) involvement is estimated to be present in 10–60% patients with pSS. The present study aimed to evaluate brainstem auditory evoked potentials (BAEP) in pSS patients without central nervous system involvement, and without otolaryngological abnormalities.

**Method:**

Thirty-six pSS patients (35 women, 1 man, mean age 48 years old) and 40 healthy volunteers were investigated. BAEP, brain imaging, laboratory parameters, and clinical neurological and otolaryngological examinations were performed.

**Results:**

Abnormal BAEP were recorded in 16.7% patients. The mean wave BAEP I and V latency and mean wave III-V and I-V interpeak latencies were significantly longer in pSS patients than the controls. There were no statistically significant correlations between BAEP parameters and laboratory tests. None of the patients was found having the abnormalities on brain imaging.

**Conclusions:**

This study confirms that in pSS patients without clinical central nervous system impairment, auditory pathway disturbances could be found. Disorders of brain bioelectrical activity may be a consequence of ongoing autoimmune process.

**Table:** 

**Key Points** *• BAEP abnormalities confirmed the clinically observed involvement of the central nervous system in patients with pSS.* *• Brain bioelectrical activity dysfunctions in pSS patients may be a consequence of ongoing inflammatory and/or immunological processes.*

## Introduction

Primary Sjögren’s syndrome (pSS) is a chronic, multisystem autoimmune exocrinopathy characterized by dysfunction of the exocrine glands. The pathogenesis of pSS is still unclear due to the heterogeneity of clinical phenotypes and pathogenetic mechanisms. Central nervous system (CNS) involvement is estimated to be present in 10–60% patients with pSS [1, 2]. Hearing damage and neuropathy of the eighth cranial nerve are very rare manifestations of pSS. They can occur in up to 25% of patients with pSS, but the exact frequency of these abnormalities is unknown [3–5].

Evoked potentials study (EP) is a precise, noninvasive, and sensitive technique that enables the evaluation of the brain’s bioelectrical activity also in patients with pSS. EPs are particularly useful in patients with mild or without clinical neurological symptoms. However, these methods have not been used so far in studies on pSS, investigating its origin and methods of its evaluation.

The aim of our study was to evaluate electrophysiological parameters of brainstem auditory evoked potentials (BAEP) in pSS patients without clinical neurological deficit, and without otolaryngological abnormalities. The analyzed parameters were correlated with the clinical data and immunological parameters.

## Materials

The study comprised 36 patients with pSS (35 women and 1 man, aged 29–65 years, mean 48) who met the American College of Rheumatology/European League Against Rheumatism (ACR/EULAR) criteria for pSS [6]. Rheumatologists examined the patients and made their diagnosis.

The exclusion criteria included the presence of any neurological, psychiatric, metabolic, and deficiency disorders; patients who were treated with medicines which change brain bioelectrical activity (e.g., neuroleptics, antiepileptic) and patients with hearing impairment and with symptoms of neurological deficit. The concomitant diseases were controlled hypertension (4 patients), lipid disturbances (4 patients), euthyroid struma (3 patients), and cholelithiasis (2 patients).

The control group consisted of 40 healthy volunteers, who were matched for age and gender to the pSS patients (38 women, 2 men, aged 28–65 years, mean 48). The neurological and rheumatological protocol and the exclusion criteria were the same as in the study group.

All the subjects gave their informed consent to participate in the study, and the project was approved by the Commission of Bioethics at the Wroclaw Medical University (number of permission: KB-357/2010).

## Methods

The patients underwent a detailed neurological examination, and their mental state screened for cognitive impairment was assessed using the Mini-Mental State Examination (MMSE) and the Clock Drawing Test (CDT). All patients underwent otolaryngological and audiological examinations and head computed tomography (CT) or magnetic resonance imaging (MRI).

Disease activity was evaluated with reference to the items included in the EULAR primary Sjögren’s syndrome disease activity (ESSDAI) and EULAR Sjögren’s syndrome patients reported index (ESSPRI) [7].

Laboratory signs of inflammation were evaluated by assessing levels of erythrocyte sedimentation rate (ESR), C-reactive protein (CRP), IgG, and C3 and C4 components. Serological evaluations included anti-Sjögren’s syndrome antigens A and B (anti-SSA and anti-SSB antibodies), anti-Ro-52 antibody, antinuclear antibody (ANA), and rheumatoid factor (RF). Hypergammaglobulinemia was measured by electrophoresis of serum and ANA antibodies measured by indirect immunofluorescence and ENA by ELISA.

The BAEP procedure was compliant with the International Federation of Clinical Neurophysiology (IFCN) [8]. EP were recorded using superficial electrodes according to the 10–20 system, with reference to linked earlobes and with a forearm earthing. We used superficial Ag/AgCl electrodes with 2 kΩ impedance, and registration was done twice to assess the reproducibility of responses.

BAEP were recorded after stimulation of left and right ear auditory stimulus presented through headphones, with a frequency of 20.3 Hz, duration of 0.1 ms, and an intensity of 65 dB above the individual hearing threshold. In each of the subjects, an individual hearing threshold was marked. For unaudited ear intensity, a masking noise of 35 dB above the hearing threshold was transmitted. Ipsilateral replies were recorded using electrodes placed on the right and left ears, with the reference electrode at the vertex and with a forearm earthing. They averaged 2000 responses in the frequency range 150–3000 Hz, and the analysis time was 10 ms. We analyze the absolute latencies of waves I, III, and V and I-III, III-V, I-V interpeaks and the amplitude of waves I and V. Prolonged interpeaks to I-III and/or III-V were considered pathological only when accompanied by the prolonged latency of interpeak I-V, and the prolonged latency of wave I, when they were accompanied by changes in the latency of further components of the auditory brainstem response.

The statistical analysis was performed using STATISTICA 11.0 PL software. The normality of distribution was verified with the Shapiro-Wilko test. If a normal distribution was stated, the groups were compared using the parametric Student *t* test. If the parameter value distributions differed significantly from a normal distribution, the non-parametric Mann-Whitney *U* test was used to compare the groups. The ANOVA test was used to compare more than 2 variables in the non-combined groups. Correlation coefficients were calculated and assessed using Pearson’s correlation coefficient. Possible sex and age influence between the study and control group were analyzed using the chi^2^ test. A *p* value < 0.05 was considered statistically significant.

## Results

There were no significant differences in age (analysis of variance; *p* = 0.13) and sex distribution (chi^2^ = 0.63, *p* = 0.92) between pSS patients and the control group.

### Rheumatological examination and laboratory parameters analysis

The disease duration was from 7 months to 27 years (mean 9.9 ± 6.4 years). The typical symptoms, physical condition, and the most common subjective complaints in pSS patients are shown in Table 1. The mean ESSDAI total score was 18 (4–25) and ESSPRI total score 5.2 (0–8). No correlation between pSS activity expressed on ASSDAI and the duration of the disease was found (*p* = 0.21).

**Table 1:** Physical condition and subjective complaints in primary Sjögren’s syndrome patients (*n* = 36)

Sicca symptoms	*n* (%)
Xerostomia and keratoconjunctivitis sicca	31 (86.1)
Lymphocytic infiltration in labial salivary gland biopsy ≥ 1	29 (80.6)
Ultrasonographic abnormalities of parotid and submandibular glands	21 (58.3)
Skin changes (purpura, urticaria)	10 (27.8)
Peripheral arthritis	10 (27.8)
Lymphadenopathy	5 (13.9)
Raynaud phenomenon	4 (11.1)
Subjective complaints	*n* (%)
Chronic fatigue	21 (58.3)
Peripheral arthralgia	21 (58.3)
Sleep disorders	17 (47.2)
Dizziness	10 (27.8)
Headache	7 (19.4)
Depressed mood (lasting ≥ 2 weeks)	6 (16.7)
Concentration difficulties	5 (13.9)

The mean value for C-reactive protein was 4.1 mg/L (0.25–9.34 mg/L), complement component C3 1.03 g/L (0.23–1.45 g/L), complement component C4 0.24 g/L (0.09–1.21 g/L), IgG 16.4 g/L (9–48 g/L), IgA 3.1 g/L (1.4–12.1 g/L), and IgM 1.3 g/L (0.5–2.5 g/L).

Further immunological and laboratory disturbances are shown in Table 2.

**Table 2:** The immunological and laboratory characteristics of primary Sjögren’s syndrome patients (n = 36)

Laboratory test	*n* (%)
RF seropositive	29 (80.6)
Anti-SSA antibody seropositive	26 (72.2)
ANA seropositive	24 (66.7)
Anti-SSB antibody seropositive	23 (63.9)
Lymphopenia < 1500/μL	22 (61.1)
Anti-Ro-52 antibody seropositive	21 (58.3)
ESR > 20 mm/h	17 (47.2)
Hypergammaglobulinemia (> 1.2 g/dL)	16 (44.4)
Leucopenia < 4000/μL	12 (33.3)
Low complement component C3 level (< 0.9 g/L)	9 (25.0)
Total protein > 8.3 g/dl	4 (11.1)
Low complement component C4 level (< 0.1 g/l)	4 (11.1)

*RF* rheumatoid factor; *anti-SSA* anti-Sjögren’s syndrome antigens A; *anti-SSB* anti-Sjögren’s syndrome antigens B; *ANA* antinuclear antibody; *ESR* erythrocyte sedimentation rate; *n* number

### Neurological examination

The neurological examination was normal in 33 patients (91.7%). In 3 cases (8.3%), there were modest symptoms of peripheral nervous system (PNS) involvement, such as attenuation of superficial sensation in the distal parts of the legs, as well as decreased ankle reflexes. None of the pSS patients was found having abnormalities on head examination. The results of the MMSE and CDT were normal in all pSS patients.

### Brainstem auditory evoked potentials

**Fig. 1:**
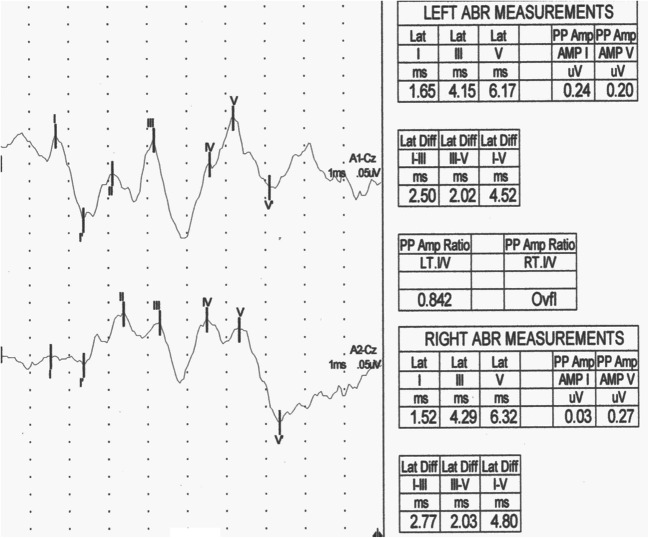
BAEP in pSS female—prolonged latencies and interpeak latencies: III (L-4,15 ms, R-4,29 ms), V (L-6.17 ms, R-6,32 ms), I-III (L-2,5 ms, R-2,77 ms), and I-V (L-4,52 ms, R-4,8 ms)

Abnormal BAEP were recorded in 6 patients (16.7%): in 3 patients were prolonged V latency and I-V interpeak latency; in 2 patients were prolonged III latency and I-V interpeak latency; and in 1 patient was prolonged III and V latencies and III-V and I-V interpeak latencies (Fig. 1).

The mean wave BAEP I and V latencies and III-V and I-V interpeak latencies were significantly longer in pSS patients compared with that of the control group (Table 3). The mean amplitude of wave I and V did not differ significantly between the two groups. Patients with symptoms of peripheral nervous system involvement had normal BAEP.

**Table 3:** Mean values of the latency (ms) and amplitude (μV) of BAEP parameters in primary Sjögren’s syndrome patients and the control group

BAEP	Patients with pSS *n* = 36			Control group *n* = 40			*p* value
	Mean	Median	SD	Mean	Median	SD	
Latency (ms)							
I	1.65	1.65	0.12	1.60	1.60	0.12	0.01*
III	3.80	3.82	0.17	3.76	3.75	0.14	0.16
V	5.77	5.77	0.23	5.64	5.64	0.21	*0.001*
I-III	2.15	2.15	0.17	2.15	2.16	0.11	0.88
III-V	1.97	1.95	0.19	1.87	1.86	0.16	*0.001*
I-V	4.12	4.08	0.23	4.04	4.03	0.18	*0.01*
Amplitude (μV)							
I	0.27	0.25	0.13	0.28	0.29	0.11	0.45
V	0.41	0.42	0.13	0.43	0.47	0.12	0.31

*BAEP* brainstem auditory evoked potentials; *pSS* primary Sjögren’s syndrome; *SD* standard deviation; *n* number

*Statistically significant changes (*p* < 0.05), Student’s *t* test

We recorded significant prolongation of the I latency and III-V and I-V interpeak latencies in pSS patients lasting more than 10 years compared with that of the patients with a shorter duration of the disease (Table 4). Disease duration had no significant impact on I and V waves amplitudes.

**Table 4:** Mean values of BAEP parameters in patients with primary Sjögren’s syndrome depending on disease duration

BAEP	Patients with pSS		Patients with pSS		*p* value
	10 years or less (*n* = 19)		more than 10 years (*n* = 17)		
Latency (ms)	Mean	SD	Mean	SD	
I	1.59	0.11	1.69	0.11	0.002*
III	3.78	0.16	3.80	0.15	0.34
V	5.73	0.22	5.82	0.20	0.14
I-III	2.14	0.17	2.16	0.17	0.94
III-V	1.89	0.15	2.02	0.15	*0.02*
I-V	4.05	0.14	4.21	0.17	*0.001*
Amplitude (uV)					
I	0.26	0.12	0.29	0.13	0.55
V	0.43	0.12	0.44	0.14	0.62

*BAEP* brainstem auditory evoked potentials; *pSS* primary Sjögren’s syndrome; *SD* standard deviation; *n* number

*Statistically significant changes (*p* < 0.05), Mann-Whitney *U* test

In pSS patients complaining about sleep disorders, chronic fatigue, and dizziness, we recorded significantly longer latencies and interpeak latencies than in pSS patients without these disturbances (Table 5).

**Table 5:** Significant changes in BAEP parameters depending on reported subjective complaints in patients with primary Sjögren’s syndrome

Complaint	Presence			Absence			*p* value
Chronic fatigue syndrome	*n* = 21			*n* = 15			
	Mean	Median	SD	Mean	Median	SD	
Latency (ms)							
V	5.83	5.83	0.23	5.72	5.73	0.21	0.001*
I-V	4.16	4.14	0.23	4.07	4.06	0.19	*0.05*
Sleep disorders	*n* = 17			*n* = 19			
Latency (ms)							
V	5.81	5.81	0.22	5.73	5.72	0.19	*0.001*
III-V	1.97	1.95	0.21	1.85	1.86	0.16	*< 0.001*
Dizziness	*n* = 10			*n* = 26			
Latency (ms)							
I	1.70	1.69	0.12	1.62	1.62	0.13	*0.05*
V	5.79	5.79	0.17	5.67	5.66	0.16	*0.001*

*SD* standard deviation; *n* number

*Statistically significant changes (*p* < 0.05)

There were no statistically significant correlations between the mean BAEP parameters and the disease activity, laboratory tests, focus score, and presence of antibodies.

## Discussion

Numerous studies have confirmed the possible involvement of the nervous system as one of the first manifestations of pSS. It is estimated that in the course of pSS, involvement of the peripheral nervous system affects 10–60% of patients and central nervous system 20% [1, 9]. The most common peripheral manifestations of pSS are axonal sensorimotor polyneuropathy, distal sensory polyneuropathy, small fibreneuropathy, and cranial nerves. Central nervous system is spilled or focal lesions of hemispheres, brainstem, cerebellum, and spinal cord [10–12]. Among the psychiatric manifestations, cognitive, depressive, anxiety, and fatigue are most often observed [10, 13]. The etiopathogenesis of nervous system damage in patients with pSS is still under investigation. Three potential pathogenic mechanisms are currently being discussed. The first assumes that neurological symptoms are a consequence of intracranial mononuclear infiltrates, the second concerns the direct, pathogenic effect of anti-neuronal and anti-Ro antibodies on the nervous system structure, and the third assumes the appearance of ischemic lesions in the course of inflammation of small vessels [11, 14].

Neuropathy of the VIII nerve in the course of pSS is described primarily as an isolated lesion [15]. A common complication of otolaryngology observed in autoimmune diseases is sensorineural hearing loss (SNHL). It is defined as a hearing loss of at least 30 dB, in three consecutive audiogram frequencies due to impairment of conduction in the VIII nerve, inner ear, or central brain processing centers [16, 17]. The etiology of this syndrome is still unclear, but a good response to glucocorticosteroids treatment and recurrent nature suggests its association with immune system disorders [16, 18]. The most commonly discussed hypothesis is based on the toxic activity of antibodies and cytotoxic apoptosis mediated by T cells in the inner ear. A single study suggests that anti-phospholipid antibody and anti-heat shock protein-70 are associated with SNHL. It has also been suggested that these autoantibodies induce thrombosis in the labyrinth vessels, thus causing damage to the inner ear resulting in SNHL [17, 19]. Boki et al. demonstrated the relationship between pSS and sensory-nervous deterioration of cochlear hearing, mainly including high frequencies [20]. In single studies, it has been demonstrated that progressive hearing loss in patients with pSS is more common than in the general population and may correlate with the level of cytoplasmic antibodies [18]. The occurrence of anticardiolipin antibodies in patients with pSS and loss was also higher than without hearing loss [10].

In the available literature, only one BAEP analysis in patients with pSS can be found. Gockay et al. analyzed a multimodal EP study in 90 patients suffering from pSS. They did not show any significant abnormalities of VEP and BAEP as well as statistically significant changes in the parameters of these potentials compared with the control group [21]. In our patients, we found significantly longer I and V wave latency and III-V and I-V interlatencies in comparison with that of the control group. The changes described by us testify to the cerebral bioelectrical activity dysfunction in patients with pSS. The changes in the BAEP components may be a reflection of subclinical auditory nerve injury, which in pSS has an autoimmune base. Longer V wave latency and III-V and I-V interlatencies in pSS patients indicate impaired transmission of pulses in the upper part of the pons and in the midbrain as well as abnormalities between the auditory nerve and the midbrain [22, 23].

Our results have shown the effect of disease length on BAEP parameters. In patients who have been ill for more than 10 years, we have found longer wave latencies and longer interlatencies of III-V and I-V BAEP compared with that of the people who have been ill for a shorter period. This phenomenon may indicate a constant negative effect of the autoimmune process and insufficient inhibition of the disease despite proper treatment. The influence of disease length on evoked potential parameters was also demonstrated in other studies. The latency of the N200 and P300 waves of endogenous cognitive potential and reduction of the amplitude of the N13/P16 somatosensory EP wave were found in patients with pSS suffering from more than 10 years of age [24, 25].

We have shown that over 80% of patients with pSS reported subjective complaints about the nervous system despite proper treatment. The most frequent complaints were chronic fatigue syndrome (58.3%), sleep disorders (47.2%), and headache and dizziness (27.8 and 19.4%), respectively.

Fatigue is one of the very frequent complaints reported in chronic and autoimmune diseases. The feeling of tiredness is usually described by patients as mental fatigue, a slower reaction to stimuli, excessive sleepiness, lack of ambition, and even depression [13, 26]. EP studies in patients with fatigue syndrome are rare. Pokryszko-Dragan et al. [27] analyzed BAEP in patients with multiple sclerosis depending on the presence and severity of chronic fatigue syndrome. In patients with fatigue syndrome in comparison with patients without such a syndrome, they showed an increase in the latency of the V wave and I-III-V interlatencies. In our study in patients who reported persistent fatigue (58.3%), we found significantly longer V wave latency and I-V interlatency compared with that of the patients who did not report this complaint. The obtained results indicate more severe brain bioelectric disorders in patients reporting fatigue.

Increased incidence of sleep disorders in autoimmune diseases is associated with the activity of the proinflammatory cytokines (TNF-α, interferon γ, IL-1, IL-2, and Il-6), which contribute to the etiopathogenesis of the underlying disease, and are also factors affecting the regulation the rhythm of sleep and wakefulness [28]. In experimental animal studies, it was demonstrated that exogenous administration of IL-1 or TNF-α results in prolonged sleep in the NREM phase and an increase in the amplitude of free waves in EEG [29]. Gudbjörnsson et al. [30] noted that in patients with pSS, very often sleep deficit and delayed falling asleep occur, caused by pain and muscle hypertension, compared with that of the control group and patients with rheumatoid arthritis. In the available literature, there are no papers analyzing EP in cases of sleep disorders in pSS. There are, however, a number of reports regarding EP in diseases whose main symptom is sleep disorders. They most often concern patients with obstructive sleep apnea (OSA, obstructive sleep apnea) [31]. In the case of BAEP in OSA patients, the prolongation of the I, V, less frequent I-III, and III-V interlatencies has been described, whereas in severe forms of the disease prolongation interlatency I-V [32]. In our study, we showed a significantly longer V wave latency and III-V BAEP interlatencies in patients who reported sleep disorders compared with patients who did not report them.

Abnormalities of BAEP parameters in patients reporting certain ailments regardless of variables associated with the disease itself may support the hypothesis of disturbed bioelectric activity due to subclinical CNS damage as their background, not just purely subjective origin.

## Conclusions

The results of the prolongation of the mean values of BAEP component latencies obtained by us confirmed the clinically observed involvement of the central nervous system in patients with pSS. Brain bioelectrical activity dysfunctions in these may be a consequence of ongoing inflammatory and/or immunological processes. The analysis of BAEP may be a useful method of assessment of the CNS in patients without clinical neurological symptoms.

## Data Availability

The numerical data used to support the findings of this study are available from the first and corresponding author upon request.
